# Analgesia for Upper Abdominal Surgery, a Scoping Review of the Current Fascial Plane Block Techniques

**DOI:** 10.3390/jcm14248632

**Published:** 2025-12-05

**Authors:** Maria T. Fernandez Martin, Edward R. Mariano, Luis F. Valdes-Vilches, Servando Lopez Alvarez, Nabil Elkassabany

**Affiliations:** 1 Department of Anaesthesiology, Rio Hortega University Hospital, 47012 Valladolid, Spain; maitefm70@hormail.com; 2 Department of Anaesthesiology, Perioperative and Pain Medicine, Stanford University School of Medicine, Stanford, CA 94305, USA; emariano@stanford.edu; 3 Department of Anaesthesiology, Costa del Sol Hospital, 29603 Marbella, Spain; lvaldes01@gmail.com; 4 Department of Anaesthesiology, Abente y Lago Hospital, 15006 A Coruña, Spain; servando.alais@gmail.com; 5 Department of Anesthesiology, University of Virginia, 1215 Lee Road, Charlottesville, VA 22903, USA

**Keywords:** intercostal nerves, regional anaesthesia, upper abdominal surgery, analgesia, TAP block, sono-anatomy, fascial plane blocks, postoperative pain management

## Abstract

Effective pain management following upper abdominal surgery, particularly in the area between the lower costal margin and the umbilicus, remains a clinical challenge. The sixth to eleventh intercostal nerves provide sensory innervation not only to this area but also to the area directly below the umbilicus, and various regional anaesthesia techniques have been described to block these nerves and reduce postoperative pain. Over the past decade, several approaches have emerged that target these nerves within the relatively confined anatomical space between the anterior axillary line and the midline. This review explores the various techniques employed to block the lower intercostal nerves, focusing on the anatomical, sonographic, and technical considerations of each technique. Traditional and contemporary approaches to providing analgesia to the upper abdominal wall will be discussed. An understanding of the differences and/or similarities of the sono-anatomy of the target fascial planes is crucial for success when performing these blocks. Further research to identify the most effective and reliable regional techniques for upper abdominal surgery is still needed.

## 1. Introduction

Optimal management of postoperative analgesia in abdominal surgeries has long been a focus of clinical research and innovation. The implementation of multimodal analgesia in now widely recognised as the best practice in clinical pain management [1], combining various analgesic techniques to achieve effective pain control, minimise opioid use, reduce adverse effects, and support enhanced recovery protocols. Historically, thoracic epidural analgesia—typically administered for 48 to 72 h with local anaesthetics or opioids—was considered the gold standard. However, with the advent of minimally invasive surgical techniques, the widespread use of anticoagulants, and the increasing prevalence of ultrasound-guided regional anaesthesia, there has been a marked decline in the use of epidurals. Consequently, the focus has shifted towards the utilisation of fascial plane blocks as a potential alternative [2].

**Figure 1:**
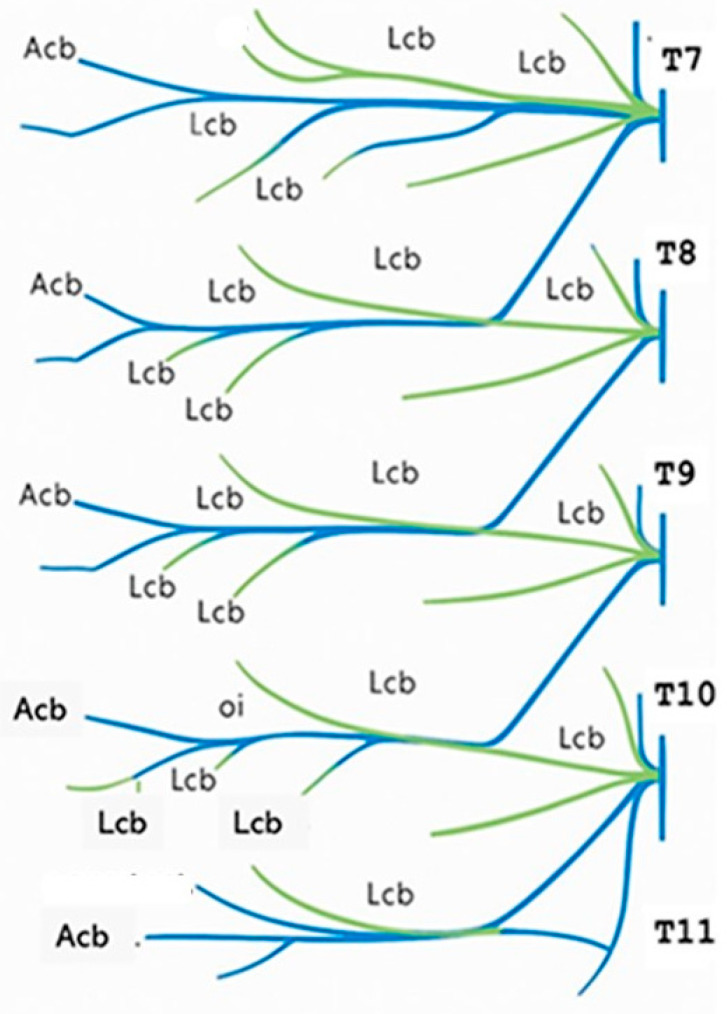
Scheme of connections between intercostal nerves and cutaneous branches. Spinal nerves (T: Thoracic); Lcb (lateral cutaneous branches); acb (anterior cutaneous branches).

Effective postoperative pain management after upper abdominal surgery, especially laparotomy, remains challenging due to the complex innervation of this region. Somatic pain originating from the upper abdominal wall is transmitted through the intercostal nerves (T7–T11) [3] (Figure 1), which are interconnected at various levels via their lateral branches., whereas visceral pain originates from the peritoneum and intra-abdominal organs. Postoperative abdominal pain is predominantly mediated by parietal somatic afferents, which account for most of the discomfort following abdominal surgery. This somatic pain can be effectively managed using fascial plane blocks, which have evolved from traditional intercostal nerve blocks [4]. These techniques typically require a single injection, offering a simplified approach compared to conventional multi-injection methods. However, in the context of minimally invasive surgery, the pain profile shifts toward visceral origins. Visceral pain is less responsive to regional anaesthesia and often necessitates systemic or neuraxial analgesic strategies for adequate control [5].

The ASRA-ESRA consensus on standardised nomenclature was prompted by the growing numbers of fascial plane blocks and the resulting confusion surrounding names and definitions [6]. Despite the publication of this consensus statement, controversies related to nomenclature persist, reflecting the ongoing challenge of achieving universal clarity and consistency in clinical and academic settings.

Intercostal nerve blocks can be performed at different points along the nerve’s path. First described by Braun in 1907, this technique evolved with Berlinkof’s 1948 account of prolonged intercostal block for upper abdominal surgery. The subcostal abdominis plane block (STAP) was described to cover surgeries in the upper abdominal wall, as an alternative technique to the traditional TAP, which typically targets the lower abdomen. Following the description of the subcostal TAP block, several other blocks have been described to provide analgesia of the upper abdominal wall. Considering the number of blocks used for this purpose, and the potential confusion that may ensue in education and clinical practice, we designed this review to report all recently published techniques for fascial plane blocks of the upper abdominal wall and critically evaluate their evidence basis.

## 2. Materials and Methods

Designed as a systematic review but presented as a scoping review due to the heterogeneity of studies, the protocol was drafted in line with Preferred Reporting Items for Systematic Reviews and Meta-Analyses (PRISMA) extension for narrative reviews guidance. The review was registered in PROSPERO CRD42023452549. The search strategy was devised by the librarian MLA, reviewed by a qualified librarian experienced in literature searches (see Supplementary Materials Section) and implemented in the MEDLINE, EMBASE, and COCHRANE databases. No time limit was set until February 2025; however, studies older than 25 years were excluded to ensure that the review focused on contemporary research. The PICO question for this review is as follows: In adults having upper abdominal surgery, does an intercostal fascial plane block (IFPB) improve postoperative pain control, lower opioid use, and reduce adverse effects compared to no nerve block or other regional anaesthesia? The search terms were ‘abdominal surgery’/exp OR ‘abdominal surgery’ OR (abdominal AND (‘surgery’/exp OR surgery) AND (‘interfascial plane block’/exp OR ‘interfascial plane block)/AND (‘fascial plane block’/exp OR ‘fascial plane block/‘intercostal nerves block)’) OR (intercostal AND nerves AND block/‘intercostal nerve block’/exp OR ‘intercostal nerve block’).

Only randomised controlled trials (RCTs) and cohort studies related to upper abdominal surgery were included.

M.T.F., and S.L.A. independently reviewed studies for relevance, and the titles, abstract, and main text were screened to select the final cohort of publications for review. The authors discussed disagreements and, if necessary, consulted L.F.V., E.M., and N.E. until they reached a consensus.

However, after completing the literature search, we found few valid articles. That is why the authors considered the evidence not good enough, without providing a synthesised conclusion. To ensure that no relevant information about these techniques was missed, we conducted a new search, focused specifically on targeting each published block. Our bibliographic search strategy consisted of searching for each block and its application in abdominal surgery. A total of 60 articles were identified, with the majority focusing on the subcostal transversus abdominis plane (TAP) block (35 articles). After removing duplicates and excluding non-clinical-trial studies, 49 articles remained eligible for review following the same criteria as for systematic review. The findings from the final selection have been structured as a historical overview.

In conclusion, we synthesised the results from a systematic review and a subsequent targeted review to deliver a comprehensive assessment of the efficacy of intercostal fascial plane block (IFPB) for postoperative analgesia in upper abdominal surgery. The results have been presented as a scoping review, a map of the evidence landscape, showing breadth rather than depth. A formal meta-analysis could not be performed due to the heterogeneity and methodological limitations of the available studies, as well as the inability to apply GRADEpro for evidence grading. Most of the randomised controlled trials (RCTs) included had small sample sizes, were conducted in single-centre settings, reported unclear blinding procedures, and had short follow-up periods. These characteristics limit the feasibility of quantitative synthesis and formal assessment of evidence certainty.

Please note that Figure 1 and Figure 4 included in this manuscript have been modified or created using Microsoft 365 Copilot. The modifications involved enhancing clarity, adjusting labelling, and refining visual elements. All modifications were performed with the aim of improving scientific communication and maintaining anatomical accuracy.

## 3. Results

The present scoping review commences with methodological considerations prior to an exploration of clinical application. The review here highlights the strengths, limitations, and potential efficacy of intercostal fascial nerve blocks on various upper abdominal surgeries.

The systematic search revealed a range of intercostal nerve blocks used for upper abdominal surgeries. Initially, 2116 articles were identified through database searching. After removing duplicate records across databases and excluding studies that did not meet the predefined eligibility criteria, a total of 13 studies were included in the final analysis.

**Figure 2:**
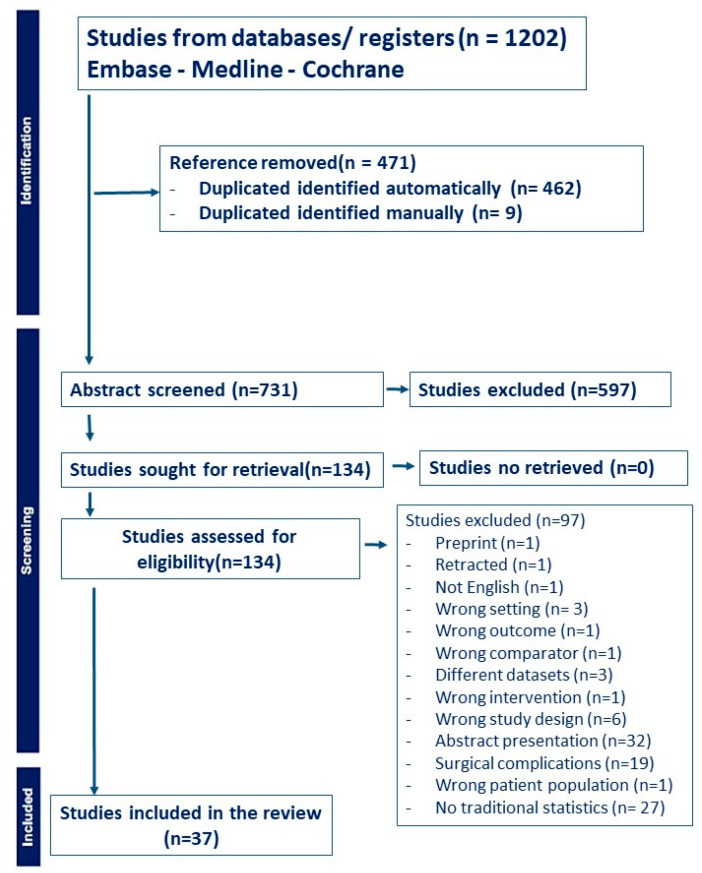
PRISMA flow diagram.

The PRISMA flow diagram (Figure 2) outlines abstract screening, full-text reviews, exclusion reasons, and the Table S1 (see Supplementary) shows preferred Reporting Items for Systematic reviews and Meta-Analyses extension for Scoping Reviews (PRISMA-ScR) Checklist. The studies included in the systematic review are included in Table 1 [7,8,9,10,11,12,13,14,15,16,17,18,19].

After conducting a secondary targeted search on each published block, consistent with the inclusion criteria applied in the systematic review, the data retrieved was systematically aggregated and temporally indexed to facilitate longitudinal analysis. The final selection of studies for this search is presented in the discussion.

**Figure 3:**
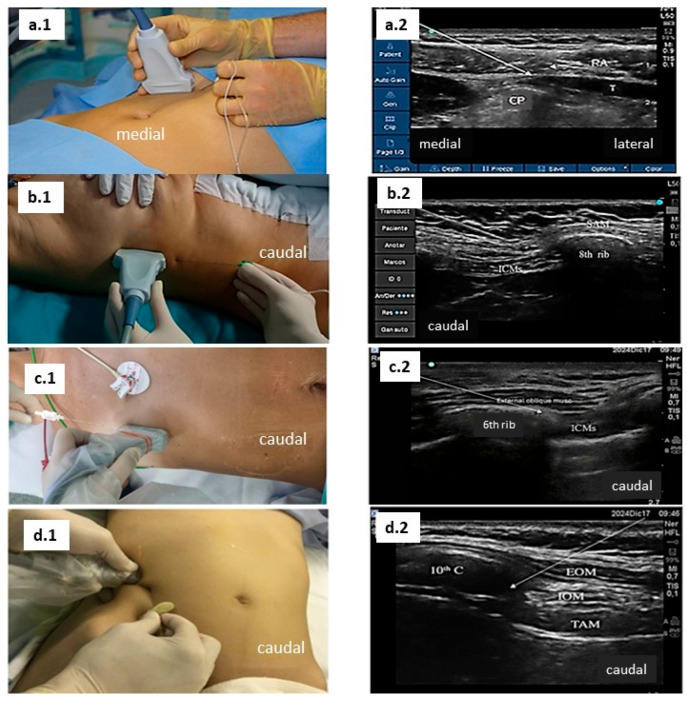
Approaches and ultrasound images: (**a.1**,**a.2**). subcostal TAP approach and ultrasound image; (**b.1**,**b.2**). SIPB approach and ultrasound image; (**c.1**,**c.2**). EIOB approach and ultrasound image; and (**d.1**,**d.2**). M-TAPA approach and ultrasound image.

In 2008, Hebbard et al. described the ultrasound-guided subcostal TAP [20] to manage pain relief in supra-umbilical surgeries. The subcostal approach to the TAP block ideally anaesthetises the intercostal nerves T6–T9. With the patient lying in the supine position, a linear transducer is placed alongside the lower margin of the rib cage as medial and cranial as possible to visualise the muscle layers of the anterior abdominal wall. The needle insertion point is near the xiphoid process (Figure 3(a.1,a.2)), and the local anaesthetic is initially deposited between transversus abdominis and the rectus abdominis muscles (Figure 3(a.1,a.2)). Subsequently, the needle is directed inferiorly and laterally to distend the transversus abdominis plane, which is parallel to the costal margin. In essence, this block could be considered a more lateral approach to the rectus sheath block, which only addresses the anterior branches of the intercostal nerves. Oblique subcostal transversus abdominis plane (OSTAP) block represents a modification of the subcostal TAP block, differing mainly in the needle trajectory, which is directed at a more oblique angle.

In 2015, an intercostal nerve block at the level of the eighth rib was described. It was named the ‘modified BRILMA’ [21] (block of the rami of the intercostal nerves in the middle axillary line), derived from the BRILMA block for breast surgery, and was later termed the low serratus–intercostal interfascial plane block (SIPB). According to the ASRA-ESRA standardised nomenclature, this technique is considered a low and deep serratus anterior plane block. This technique has been described as an analgesic option for patients who experience the conversion of a planned laparoscopic cholecystectomy to an open procedure. The block is performed with patients in a supine position. The US transducer is to be placed following the middle axillary line, with the needle being advanced in plane caudo-cranially (Figure 3(b.1,b.2)). When the needle tip reaches the fascial plane between the serratus anterior muscle and the external intercostal muscle at the eighth rib, a 15 m bolus dose of local anaesthetic is administered, observing the spread in the fascial plane with ultrasonography (Figure 3(b.1,b.2)).

Hamilton et al. reported in 2019 [22] that staining of only the lateral cutaneous branches of T6–T11 resulted from injection at the T6 level superficial or deep to the external oblique muscle around the midclavicular line. Tulgar et al. described the thoraco-abdominal perichondrial approach (TAPA) in 2019, where local anaesthetic is injected deep to the external oblique and superficial to the costal cartilage, with a second injection deep (posterior) to the costal cartilage between the internal oblique and transversus abdominis origins. This block covered T5–T6 dermatomes from the anterior axillary line to 4–5 cm lateral to the sternum as well as T7–T12 dermatomes from the anterior axillary line to the midline. The TAPA block aims to target the anterior branches of the thoracic–abdominal nerves. Following the description of TAPA, the same group redefined the TAPA block and named this novel technique as modified-TAPA (M-TAPA) [23]. This modification was described to target certain dermatomes based on the surgical incision sites. The linear probe is placed on the costochondral angle in the sagittal plane under ultrasound guidance at the 10th costal margin. A deep angle is given to the costochondral angle at the edge of the 10th rib with the probe in the sagittal direction to view the lower surface of the costal cartilage in the midline. The needle is inserted in the cranial direction using the in-plane technique and the needle tip is moved to the posterior aspect of the 10th costal cartilage. A 50 mL dose of local anaesthetic (LA) is injected at the lower surface of the perichondrium (Figure 3(d.1,d.2)).

In 2021, Elsharkawy et al. [24] described the external oblique intercostal block (EOIB), which was a modification of the original report described by Hamilton in 2019. This block aimed to provide analgesia for the upper midline and lateral abdominal wall, particularly for cases in which the upper abdominal wall layers are disrupted during surgery. Injections were performed with the subjects in the supine position, depositing local anaesthetic solution between the external oblique and intercostal muscles. A linear ultrasound transducer (12–15 MHz) is placed in the sagittal plane between the midclavicular and anterior axillary lines at the level of sixth rib, with the orientation marker directed cranially. Rib six was identified either by placing the ultrasound transducer at the level of the lower costal margin, where the tenth rib is identified, and then counting, or by identifying the seventh rib at the level of the xiphoid process and then moving the transducer one rib up (Figure 3(c.1,c.2)).

Despite these different names and approaches, these fascial plane blocks all target the same region of the upper abdominal wall innervated by lower intercostal nerves.

## 4. Discussion

Following the historical evolution of all these blocks, the question remains to be answered: how are these blocks are similar and/or different from one another?

**Figure 4:**
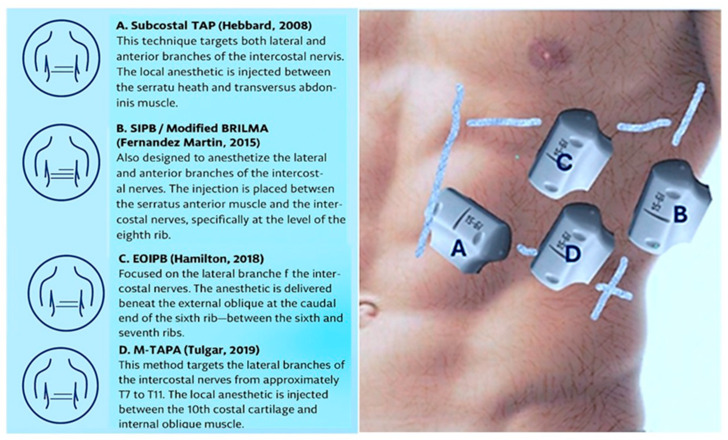
Schematic presentation of the position of the transducer during performance of the various blocks [20,21,22,23].

Our first point will be the surface approach. The transducer can be placed from lateral to medial between the mid-axillary line and the costo-condral junction line and from cephalic to caudal between the sixth rib and the subcostal line, As seen in Figure 4. The area of probe placement for the various blocks does not exceed five square inches.

**Figure 5:**
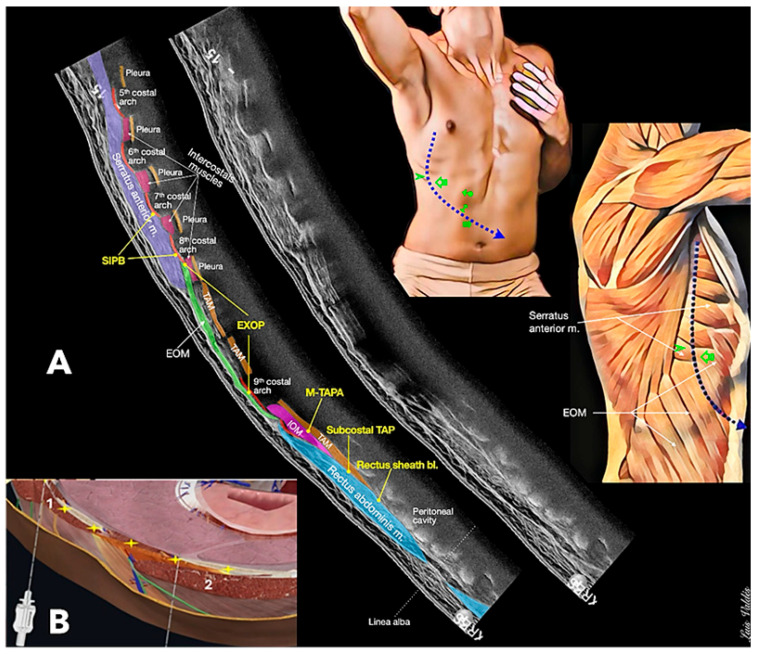
(**A**). A panoramic ultrasound scan was performed from the mid-axillary line, at the level of the 5th right costal arch to the left supra-umbilical region. The ultrasound image illustrates the close anatomical relationship between various regional anaesthesia techniques described in the literature regarding the proximity, minimal distance, and nearly identical injection planes among the M-TAPA, subcostal TAP, and rectus sheath plane blocks. Similarly, the shared injection plane and proximity of the deep serratus plane block (SIPB) and the external oblique plane block (EOIB), especially when both are performed at the level of the eighth costal arch, can be observed. (**B**). Note the interrelationship between both muscles, with the insertions of the serratus muscle interdigitating with the origin of the external oblique muscle, suggesting that the anatomical and functional similarities of these fascial planes are likely to produce comparable patterns of local anaesthetic spread. TAM: transversus abdominis muscle; IOM: internal oblique muscle; EOM: external oblique muscle. (**B**). 1: serratus anterior muscle; and 2: external oblique muscle. +: deep serratus plane block; +: external oblique plane block.

Secondly, the fascial plane to which the local anaesthetic spread is the same in some of these blocks (Figure 5). In subcostal TAP, the needle is placed in the plane between the transversus abdominis and rectus muscles, just under the rectus (15–20 mL), and in M-TAPA, the fascial plane is between the internal oblique and transversus muscles at the 10th cartilage (40–50 mL), which one could argue is the same fascial plane, especially if the recommendation is to inject a much higher volume to aid the spread of the local anaesthetic. These high volumes lead us to reflect on if high volumes (like the 40–50 mL often used in M-TAPA) increase systemic absorption risk. Frail or low-weight patients have lower thresholds for toxicity. Also, elderly or frail patients often have reduced hepatic and renal clearance, prolonged local anaesthetic half-life, and increasing toxicity risk. In contrast, patients with obesity may require higher volumes for effective spread, but this must be balanced against toxicity risk. Therefore, this is something to keep in mind when choosing a block.

To perform the serratus intercostal plane block, the local anaesthetic is placed in the fascial plane between the serratus anterior and intercostal muscles, above the eighth rib (15–20 mL); this is, essentially, a low deep serratus. Additionally, in the external oblique intercostal block, the fascial plane is between the external oblique and intercostal muscles at 6–7th rib (20 mL). Looking at the anatomy of the lateral and anterior thoracic wall, we can identify two muscles, serratus anterior and external oblique, just above the ribs, so we are talking about the same fascial plane, separated only by two cephalic ribs. Typically, injectate spread is only visualised at the time of block performance, so it is highly likely that local anaesthetic continues to spread within these adjacent planes after the block is completed.

From this perspective, the subcostal TAP and MTAPA are very similar, and SIPB and EOIB have similar fascial planes, over the rib, separated by few centimetres. As we can see in the scheme (Figure 4), the difference is only that the MTAPA is more lateral and the EIOB is more medial than SIPB.

The third point of interest is the effectiveness of these fascial blocks. The efficacy of intercostal neve blocks has been long established for analgesia for upper abdominal surgery, even compared with epidural analgesia [25]. In the search for a comprehensive approach for fascial blocks involving intercostal nerves, we found that four fascial blocks were described. The studies identified in the specific literature search focusing on “each block” and “abdominal surgery” were predominantly related to laparoscopic cholecystectomy, accounting for 20 articles. The most frequently reported block was the subcostal TAP block [26], possibly due to its status as one of the earliest techniques described. The results of this search are summarised in Table 2.

Several studies focused on evaluating the efficacy of these blocks in laparoscopic surgery, even though it may be the least painful type of surgery. Although the subcostal TAP block is the most studied technique in laparoscopic cholecystectomy, the results remain contradictory. Some authors [9,27,28,29] have reported opioid-sparing effects, improved pain control, and enhanced respiratory function. In contrast, other authors [30,31,32] do not observe any significant improvement in quality of recovery or analgesia. Furthermore, when compared with more recently developed blocks, the outcomes of the subcostal TAP block appear less favourable [33,34,35]. SIPB, EOIB, QLB, and M-TAPA have also demonstrated favourable results in terms of quality of recovery and analgesia, both when compared to control groups and to the subcostal TAP block [11,17,36,37,38].

Laparoscopic gastric surgeries represent the second-most-frequently studied category among laparoscopic procedures. Within this context, the subcostal transversus abdominis plane (TAP) block has not demonstrated significant advantages over the posterior subcostal TAP (STAP) block in terms of postoperative morphine consumption, with both groups typically requiring approximately 7 mg [39]. Furthermore, when compared to local infiltration techniques, as investigated by Albrecht [40] or Coskim [41], as well as to other fascial plane blocks [42,43,44], the subcostal TAP block similarly does not appear to confer substantial postoperative analgesic benefit. However, the serratus intercostal plane block (SIPB) [12] and external oblique intercostal block (EOIB) [15] have shown promise as effective analgesic alternatives and may be valuable components of a multimodal analgesic strategy.

SIPB has been evaluated and compared to the quadratus lumborum block (QLB) in laparoscopic nephrectomy, with both blocks showing only modest benefits [45].

Open surgery, which is typically associated with moderate-to-severe pain and presents greater challenges for anaesthesiologists, was reported in only 12 articles. The STAP technique has been shown to reduce opioid consumption both intraoperatively and postoperatively in open liver surgery [47]. Kitlik [48] and Maeda [49] reported advantages of using STAP as an analgesic technique, with 40 mg of postoperative morphine consumption or an intraoperative fentanyl use; however, Assefi [50] did not observe similar benefits in living liver donors. When compared to ESPB, STAP was found to be less effective [51]. The low serratus anterior plane block (SAPB) [52] significantly reduced pain post-surgery and reduced analgesics at 24 h post-surgery compared to the control group. Patients with continuous low SAPB also had higher global QoR-15 scores at 48 h post-surgery. Yi et al. [53] also demonstrated favourable outcomes with EOIB. In the context of open nephrectomy, STAP, QLB [54], and SIPB [10] have all provided adequate analgesia, with SIPB showing the most significant opioid-sparing effect.

The best outcomes have been achieved in supra-umbilical laparotomy, possibly because the primary pain is somatic, which is well covered by all these blocks. Subcostal TAP [55] showed even better results than epidural analgesia and SIPB in ventral hernia repair [10]. EOIB, in the published studies, presented an adequate alternative to STAP [56], prolonging the duration of PCA activation. Compared to epidural, EOIB [57] provided superior and prolonged analgesia versus IV morphine as a supplement to epidural analgesia. Additionally, the continuous SIPB was associated with a better analgesic profile compared with the control group after upper abdominal surgeries [58].

**Figure 6:**
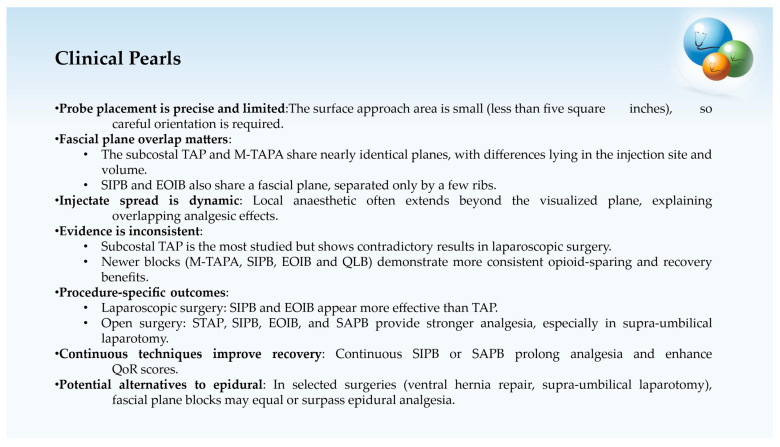
Clinical pearls.

Figure 6 summarises the key messages of the review.

Clinical relevance: Fascial plane blocks share anatomical pathways, often yielding similar spread and effects. While subcostal TAP remains foundational, SIPB, EOIB, and M-TAPA are emerging as more reliable options for multimodal analgesia in abdominal surgery, with continuous techniques offering sustained benefit.

Risk of bias: This review is limited by the fact that a meta-analysis could not be performed due to the characteristics of the available studies and the inability to apply GRADEpro. Most of the randomised controlled trials included small sample sizes, were single-centre in nature, lacked clear blinding, or had short follow-up periods. These factors restrict the strength of the conclusions and highlight the need for larger, methodologically robust trials.

## 5. Considerations for Future Research and Practice

We have presented four variations of abdominal fascial plane blocks that target the distal intercostal nerves in the medial upper abdomen, which is approximately the size of one hand. It is reasonable to question the need for multiple approaches and the importance of giving them different names. While all published reports suggest that these techniques may be effective for analgesia, the mechanisms of action of fascial plane blocks are still not fully understood. An emphasis of the ASRA-ESRA nomenclature standardisation projects was defining the “block” not as the site of needle entry but the eventual target location for local anaesthetic deposition; ultimately, the “block” should be the clinical effect of the local anaesthetic. A limitation of the existing literature is that many fascial plane block studies involve cadavers or a brief visualisation of local anaesthetic injection during clinical cases.

Of the block techniques reviewed in this article, the only one included in the ASRA-ESRA nomenclature is the subcostal TAP [3]. The other fascial plane block techniques have not yet been harmonised in the nomenclature process, and this narrative review suggests that they should be included in the next phase. The use of multimodal analgesia and the expansion of minimally invasive surgical techniques will make it difficult to demonstrate clinically important differences between fascial plane blocks. Published studies demonstrate that analgesia for laparoscopic surgery can be achieved using any of the approaches; however, STAP, EOIB, and SIPB have shown promising results in open supra-umbilical surgery. Further investigation is required to establish the efficacy of the newer blocks in open surgery beyond cholecystectomies. Studying the eventual local anaesthetics spread over time in actual patients is needed to truly understand the similarities and differences between these procedures. Given the results of the current review, perhaps more studies comparing the techniques with each other are needed to enable a more concrete evaluation of effectiveness.

For now, anaesthesiologists may choose the one that they are most comfortable performing and that is appropriate for their patients who undergo upper abdominal surgery. However, further studies are warranted to evaluate the safety and efficacy of these blocks in specific patient populations, including those undergoing bariatric surgery, laparoscopic procedures, and supra-umbilical open surgeries.

## Figures and Tables

**Table 1:** Results of the systematic review.

Author Year	Study (N° Patients)	Fascial Block	Surgery	Results
Hye-Won Jeong [7] (2019)	RCT (200)	Rectus Sheath pre/post	Laparoscopic Cholecystectomy	Pre-RSB less fentanyl at 24 h (210/267 µg) (0.02)
Chen CK [8] (2013)	RCT (40)	OSTAP/Control	Laparoscopic Cholecystectomy	OSTAP: Shorter extubation time (0.021) - VAS 2/2 (0.29)
Oksar M [9] (2016)	RCT (60)	OSTAP/TAP/Control	Cholecystectomy	OSTAP < TAP < Control VAS (0.01)
Fernandez MT [10] (2021)	RCT (102)	SIPB/Control	Upper Abdomial Surgery (Open)	SIPB: Efficient analgesia: - Morphine 24 h: 4/40 mg (0.0001) - NRS 1/4 (<0.005); QoR15 122/100
Saravanan [11] (2021)	RCT (60)	Modified BRILMA/STAP	Laparoscopic Cholecystectomy	Modified BRILMA (SIPB) = subcostal TAP - Morphine 5.3/5 mg (0.31) - VAS (p no significant)
Kara Y [12] (2023)	COHORT (30)	Modified BRILMA/Port	Bariatric Surgery	Modified BRILMA alternative to port LA - 12 h VAS lower - Opiod (0.66)
Amin [13] (2024)	RCT (63)	EOIB/STAP/Control	Supra-Umbilical Surgeries	- VAS: EOIB = subcostal TAP/control (<0.001) - Fentanyl: EOIB/STAP (0.04)
Rajitha A [14] (2024)	RCT (100)	EOIB/STAP	Laparoscopic Upper Abdominal Surgeries	EOIB is superior to STAP - NRS (<0.001) - Morphine: 1.06/3.52 mg (<0.0001)
Doymus [15] (2024)	RCT (70)	EOIB/Port	Laparoscopic Sleeve Gastrectomy	EOIB/port: 12 h VAS 3.9/4.7 (>0.08) - Fentanyl 24 h: 505/880 µg (<0.001)
Gangadhar V [16] (2024)	RCT (70)	EOIB + RS/LIA	Laparoscopic Cholecystectomy	EOIB + RSB superior to LIA: pain scores and rescue analgesia (<0.001)
Mehmet C [17] (2024)	RCT (73)	EOIB/OSTAP	Laparoscopic Cholecystectomy	EOIB = OSTAP block: VAS scores and first analgesia (>0.05)
Ozel ES [18] (2024)	RCT (60)	EIOB/Control	Laparoscopic Sleeve Gastrectomy	EOIB/control - Morphine 24 h: 10.6/24.9 mg (<0.001) - Nausea (<0.001)
Ciftci [19] (2025)	RCT (60)	MTAPA/EOIB	Laparoscopic Cholecystectomy	M-TAPA = EOIB NRS 24 h (0/0) - Morphine lower EOIB: 5/12 mg (<0.05)

**Table 2:** Summary of articles selected in the second search.

Surgery	Block	Author	Sample Size	Results
**Cholecystectomy**	-STAP	Shin et al. [27]	53 p	STAP block better analgesia than the TAP block
	-STAP	Basaran et al. [28]	76 p	Significant improvement in respiratory function and better pain relief
	-STAP	Oksar et al. [9]	60 p	STAP effective analgesic technique
	-STAP	Emile et al. [29]	110 p	Improves recovery (pain relief, PONV…)
	-STAP	Jung et al. [30]	38 p	Does not improve the quality and analgesia
	-STAP	Di Mauro et al. [31]	60 p	No additional benefit
	-STAP	Houben et al. [32]	60 p	Does not improve the analgesia provided
	-STAP/ESPB	Altıparmak [33]	76 p	ESPB more effective than STAP
	-STAP/ESPB	Ozdemir et al. [34]	64 p	ESPB provides superior analgesia
	-USicns/STAP	Xu et al. [35]	64 p	US icns T6-11 better results
	-SIPB/STAP	Saravanan et al. [11]	60 p	SIPB is equally efficacious than STAP
	-STAP/QLB	Baytar et al. [36]	107 p	Similar results. STAP easier to perform
	-EOIB/STAP	Mehmet et al. [17]	70 p	Similar analgesic activity
	-MTAPA	Güngör et al. [37]	60 p	Superior analgesia compared to infiltration
	-EOIB	Korkusuz [38]	80 p	Minimal clinically important differences
**Laparoscopic** **Gastric surgery**	-STAP	Ari et al. [39]	40 p	STAP effective analgesic technique
	-STAP	Albrecht el al [40]	70 p	Bilateral STAP blocks no analgesic benefit
	-STAP	Coşkun et al. [41]	45 p	STAP not better than infiltration
	-QLB/STAP	Nie et al. [42]	60 p	QLB provided greater opioid-sparing effect
	-ESPB/STAP	Abdelhamid et al. [43]	66 p	ESPB lowers postoperative pain scores
	-ESPB/STAP	Mu et al. [44]	168 p	ESP’s ability to reduce opioid use and promote faster recovery
	-SIPB	Kara et al. [12]	30 p	SIPB is an alternative technique
	-EOIB	Doymus et al. [15]	60 p	EOIB can be used as part of multimodal analgesia
**Laparoscopic nephrectomy**	-SIPB/QLB	Fernandez et al. [45]	120 p	SIPB and QLB showed adequate postoperative pain control
**Radical gastrectomy**	-STAP/epidural	Wu et al. [46]	90 p	Epidural better analgesia than STAP, however STAP better analgesia than opioids iv.
**Liver surgery**	-STAP	Erdogan et al. [47]	49 p	STAP reduces opioids consumption
	-STAP	Kitlik et al. [48]	50 p	STAP reduces postoperative morphine consumption
	-STAP	Maeda et al. [49]	32 p	Less intraop fentanyl consumption
	-STAP	Assefi [50]	132 p	A small opioid reducing effect after orthotopic liver transplantation surgery.
	-ESPB/STAP	Mostafa et al. [51]	60 p	ESPB provided superior analgesia
	-SIPB	Jiang et al. [52]	136 p	SIPB effective analgesia, saving opioids
	-EOIB	Yi et al. [53]	48 p	EOIB significantly enhances postoperative analgesia
	-SIPB	Fernandez et al. [10]	58 p	Effective analgesia, saving opioids
**Open nephrectomy**	-STAP/QL	Saleh et al. [54]	48 p	QLB better results than STAP
	-SIPB	Fernandez et al. [10]	15 p	Effective analgesic technique
**Ventral hernia repair**	-SIPB	Fernandez et al. [10]	26 p	SIPB provide effective analgesia and better postoperative recovery
**Supraumbilical incision**	-STAP	Niraj et al. [55]	62 p	Superior analgesia STAP to epidural
	-EOIB/STAP	Shrey [56]	50 p	EOIB effective analgesia
	-EOIB/epidural	Srinivasaraghavan [57]	66 p	EOIB alternative to epidural
	-SIPB	Mamoun et al. [58]	60 p	SIPB provides a good analgesia

STAP (subcostal transversus abdominis block), EOIB (external oblique intercostal block), SIPB (serratus intercostal plane block), QLB (quadratus lumborum block), and p (patients).

## Data Availability

Not applicable.

## Supplementary Materials

The following supporting information can be downloaded at: https://www.mdpi.com/article/10.3390/jcm14248632/s1, Table S1: Preferred Reporting Items for Systematic reviews and Meta-Analyses extension for Scoping Reviews (PRISMA-ScR) Checklist [59].

## Abbreviations

The following abbreviations are used in this manuscript:

T	thoracic
Lcb	lateral cutaneous branches
acb	anterior cutaneous branches
ASRA-ESRA	American society regional anaesthesia–European society regional anaesthesia
STAP	subcostal abdominis plane block
OSTAP	oblique subcostal transversus abdominis plane
IFPB	intercostal fascial plane block
‘modified BRILMA’	block of the rami of the intercostal nerves in the middle axillary line
SIPB	low serratus–intercostal interfascial plane block
TAPA	the thoraco-abdominal perichondrial approach/MTAPA modified the thoraco-abdominal perichondrial approach
LA	local anaesthestic
EOIB	external oblique intercostal block
QLB	quadratus lumborum block
p	patients
PCA	patient control activation
IV	intravenous
